# The Presence and Potential Role of ALDH1A2 in the Glioblastoma Microenvironment

**DOI:** 10.3390/cells10092485

**Published:** 2021-09-20

**Authors:** Stephanie Sanders, Denise M. Herpai, Analiz Rodriguez, Yue Huang, Jeff Chou, Fang-Chi Hsu, Darren Seals, Ryan Mott, Lance D. Miller, Waldemar Debinski

**Affiliations:** 1 Department of Cancer Biology, Wake Forest School of Medicine, Winston Salem, NC 27157, USA; svsander@wakehealth.edu (S.S.); dgibo@wakehealth.edu (D.M.H.); yhuang@wakehealth.edu (Y.H.); jchou@wakehealth.edu (J.C.); ldmiller@wakehealth.edu (L.D.M.); 2 Brain Tumor Center of Excellence, Wake Forest Baptist Medical Center Comprehensive Cancer Center, Winston Salem, NC 27157, USA; 3 Department of Neurosurgery, Jackson T. Stephens Spine and Neuroscience Institute, University of Arkansas for Medical Sciences, Little Rock, AR 72205, USA; arodriguez@uams.edu; 4 Winthrop P. Rockefeller Cancer Institute, University of Arkansas for Medical Sciences, Little Rock, AR 72205, USA; 5 Department of Biostatistics and Data Science, Wake Forest School of Medicine, Winston Salem, NC 27157, USA; fhsu@wakehealth.edu; 6 Biology Department, Appalachian State University, Boone, NC 28608, USA; sealsdf@appstate.edu; 7 Department of Pathology, Wake Forest School of Medicine, Winston Salem, NC 27157, USA; rmott@wakehealth.edu

**Keywords:** glioblastoma, low-grade glioma, macrophages, retinoic acid, ALDH1A2, monocytic cells, single-cell sequencing, tumor microenvironment, tumor-associated macrophages, MMP, invasion, progression, enzyme

## Abstract

Glioblastoma (GBM) is the most aggressive malignant glioma. Therapeutic targeting of GBM is made more difficult due to its heterogeneity, resistance to treatment, and diffuse infiltration into the brain parenchyma. Better understanding of the tumor microenvironment should aid in finding more effective management of GBM. GBM-associated macrophages (GAM) comprise up to 30% of the GBM microenvironment. Therefore, exploration of GAM activity/function and their specific markers are important for developing new therapeutic agents. In this study, we identified and evaluated the expression of ALDH1A2 in the GBM microenvironment, and especially in M2 GAM, though it is also expressed in reactive astrocytes and multinucleated tumor cells. We demonstrated that M2 GAM highly express ALDH1A2 when compared to other ALDH1 family proteins. Additionally, GBM samples showed higher expression of ALDH1A2 when compared to low-grade gliomas (LGG), and this expression was increased upon tumor recurrence both at the gene and protein levels. We demonstrated that the enzymatic product of ALDH1A2, retinoic acid (RA), modulated the expression and activity of MMP-2 and MMP-9 in macrophages, but not in GBM tumor cells. Thus, the expression of ALDH1A2 may promote the progressive phenotype of GBM.

## 1. Introduction

Glioblastoma (GBM), a WHO grade IV astrocytoma, is the most common and aggressive malignant glioma and carries a prognosis of only 15–17 months median survival [1,2]. Effective targeting of GBM tumors is challenging due to their heterogeneity, resistance to radiation and chemotherapy, and diffuse infiltration into normal brain parenchyma [3,4,5]. Alternatively, targeting the cells that make up the GBM tumor microenvironment and support GBM growth, progression, and immune evasion may prove an effective aid to the elimination of GBM tumors [6,7]. 

Glioblastoma-associated macrophages (GAM) comprise up to 30% of the GBM microenvironment and are thought to facilitate tumor growth, infiltration and recurrence; therefore, they represent an attractive non-transformed target for GBM therapy [8]. Macrophages are generally divided into two different activation states, namely, classically differentiated M1 macrophages and alternatively differentiated M2 macrophages. These functional states are determined by various signals within the microenvironment; M1 macrophages are stimulated by interferon gamma (IFNγ) and lipopolysaccharide (LPS) while M2 macrophages are stimulated by interleukin 4 (IL-4), IL-13, and corticosteroid hormones [9,10,11,12]. Recent evidence suggests that macrophages exist along a spectrum ranging from M1 to M2 macrophages, making even the macrophage population in tumors quite heterogeneous [11,13,14,15]. There are several markers associated with each macrophage phenotype, though no ideal marker exists. The markers for M1 macrophages can be STAT1 phosphorylation, as well as TNF, CCL5 and CXCL9 secretion [11]. The markers for M2 macrophages are CD204, CD206, and CD163 expression along with IL-10 and CCL4 secretion [11,16]. M1 macrophages primarily produce pro-inflammatory cytokines and mediate innate immune responses via antigen presentation [17]. However, the GBM microenvironment promotes GAM of the M2, anti-inflammatory phenotype, which are present in many established tumors as they promote tumor progression, tissue remodeling, and angiogenesis [18,19]. GAM express genes related to wound healing and immune suppression, along with MHC-II and costimulatory signaling genes, which suggests that GAM, at least in part, are present in a chronic wound-healing state [20]. 

GAM have been reported to contribute to the malignancy and progression of gliomas through various mechanisms including increasing GBM invasion, suppression of anti-tumor immunity, and angiogenesis [21]. For example, the expression of matrix metalloproteinase 2 (MMP-2) cleavage enzyme, MT1-MMP, has been positively correlated with increasing glioma malignancy grade [22], and GAM have also been shown to induce MMP-9 expression via release of transforming growth factor beta (TGF-β) [21,22]. Both metalloproteinases are associated with increases in glioma cell invasiveness [21]. As GAM have been shown to contribute to GBM growth and progression in various ways, it is therefore important to explore and understand the different pathways which GAM utilize to support this malignant phenotype in order to find new targets for therapy and increase the efficacy of current therapeutic strategies.

Aldehyde dehydrogenases are represented by the aldehyde dehydrogenase (ALDH) superfamily of 19 genes in the human genome [23]. Among this super family is the ALDH1 family of genes, of which ALDH1A1, -1A2, and -1A3 are the most commonly represented in human tissue [23]. ALDH1A1, -1A2, and -1A3 have differing tissue distributions but they each catalyze the synthesis of retinoic acid (RA) from retinaldehyde [24]. RA is known to play a role in many different cellular pathways including cell growth, differentiation, immunological tolerance, and adaptive immune responses [25,26]. RA has also been used as a therapeutic agent in the treatment of GBM with a limited and inconsistent effect [26]. For example, treatment with RA led to inhibited proliferation and migration of GBM stem-like cells [27,28]. However, single-agent treatment with RA had no effect in recurrent GBM; RA in combination with temozolomide (TMZ) produced partial responses only in 5% of patients [26].

In this study, we demonstrate that the ALDH1A2 is associated with M2 macrophages in a monocytic cell model system and may represent a marker for a subset of M2 GAMs. However, the enzyme is not restricted to macrophages, since it is also expressed in microglia, reactive astrocytes, and some tumor cells. We sought to explore the potential function of ALDH1A2 and the effect RA has on both GBM and macrophage cells. The results depict a complex picture of relationships that shed more light on the functioning of the GBM microenvironment.

## 2. Materials and Methods

### 2.1. Cell Culture 

THP1, T98G, U87, and U-251 MG cells were obtained from the American Type Culture Collection (ATCC; Manassas, VA, USA). THP1 cells were grown at a density of 2–8 × 10^5^ cells/mL in Mφ culture media: RPMI-1640 (Invitrogen, Waltham, MA, USA) supplemented with 10% fetal bovine serum (FBS), 2-mercaptoethanol (Gibco), and 5 units/mL penicillin–streptomycin solution (Thermofisher Scientific, Waltham, MA, USA). U-251 cells were maintained in DMEM with 10% FBS, and 0.1 mmol/L MEM nonessential amino acids (Sigma-Aldrich, St Louis, MO, USA). Patient-derived primary GBM cell lines BTCOE 4795, BTCOE 4536, BTCOE 4637, BTCOE 4810, BTCOE 4764, and G48a were generated as previously described [29,30] and maintained as sub-confluent cultures in Explant media: RPMI-1640 supplemented with 10% FBS and 4.5 g/L glucose. All patient samples used for Western blots and immunostaining were obtained from the Brain Tumor Center of Excellence (BTCOE) in the Wake Forest Baptist Medical Center Comprehensive Cancer Center and handled according to Wake Forest IRB protocol #8427. The BTCOE cell lines were authenticated back to the original patient tumor by IDEXX Bioanalytics (IDEXX Bioanalytics, Columbia, MO, USA) using a 16 STR markers profile.

### 2.2. Antibodies

Mouse monoclonal ALDH1A2 (G2), mouse monoclonal CD68 (KP-1), rabbit polyclonal CD163 (H-130), mouse monoclonal CD204/SR-A (E-5), goat polyclonal CD206 (C-20), and goat polyclonal GFAP (C-19) antibodies were purchased from Santa Cruz Biotechnology (Santa Cruz Biotechnology, Dallas, TX, USA). Mouse monoclonal anti-β-Actin (AC-15) was purchased from Sigma-Aldrich (Sigma-Aldrich, St Louis, MO, USA). Rabbit polyclonal anti-MMP-7 and rabbit monoclonal anti-CD163 (EPR19518) were purchased from Abcam (Abcam, Waltham, MA, USA). Mouse monoclonal CD163 (5C6-FAT) was purchased from Novus Biological (Novus Biological, Littleton, CO, USA). Mouse monoclonal anti-ALDH1A1 (1G6) was purchased from Abnova (Abnova, Walnut, CA, USA); mouse monoclonal anti-ALDH1A3 (OTI4B6) was purchased from Invitrogen (Invitrogen, Waltham, MA, USA). 

### 2.3. Polarization of Macrophages

THP1 cells were seeded into 6-well plates at a density of 10^6^ cells/well. Phorbol myristate acetate (PMA; Sigma, St Louis, MO, USA; 40 nM;) was added to M0, M1, and M2 conditions and all cells were incubated for a total of 48 h at 37 °C. For polarization, 100 ng/mL lipopolysaccharide and 20 ng/mL interferon-γ (Peprotech, Rocky Hill, NJ, USA; M1), or 20 ng/mL IL-4 and 20 ng/mL IL-13 (M2) (made in our laboratory), were added 6 h after activation with PMA [31,32]. 

### 2.4. Glioma Conditioned Media Assay 

U-251 MG or patient-derived GBM cell lines were cultured in 6-well plates for 48 h at 37 °C in serum-free RPMI-1640 + 2-mercaptoethanol at a density of 10^6^ cells/well. At 48 h, media were removed and centrifuged to remove debris. Conditioned media (CM) were stored at −80 °C until use. THP1 cells were seeded into 6-well plates at a density of 10^6^ cells/well in one of three culture conditions: Explant media, Mφ culture media, or 1:1 Mφ culture media + CM. Cells were cultured for 48 h at 37 °C then harvested for protein collection.

### 2.5. Zymograms

A total of 3 × 10^5^ U-251, BTCOE 4536, 4795, or 4810, or naïve THP1 cells were cultured in serum-free RPMI-1640 either as monocultures or as co-cultures with 3 × 10^5^ GBM and 3 × 10^5^ THP1 cells in 6-well plates. Relevant cultures were treated with RA at increasing concentrations and cultures were subsequently protected from light. After 48 h, CM was collected, centrifuged to remove debris, and stored at −80 °C until use. Gelatin zymography was performed as previously described [33]. 

### 2.6. Cell Migration Assay

U-251 cells were grown to a confluent monolayer of cells in a 6-well plate in Mφ culture media. For each condition, three scratch wounds were made using a sterile 200 μL tip and cells were rinsed with sterile PBS to remove detached cells. For co-culture conditions, 5 × 10^5^ THP1 cells were then added to the culture well. Phase-contrast microscopy pictures were taken of the same field at 0 h and 16 h and ImagePro Plus (ver 9.1, Media Cybernetics, Silver Springs, MD, USA) software was used to calculate the width of scratch at each time point in order to calculate the distance travelled by U-251 cells.

### 2.7. Microarray Analysis

THP1 cells were differentiated and polarized as described above. At 48 h, RNA was isolated using the PureLink RNA Mini Kits (Invitrogen, Carlsbad, CA, USA) according to the manufacturer’s instructions and submitted to the Cancer Genomics Shared Resource in the Wake Forest Baptist Medical Center Comprehensive Cancer Center. RNA was analyzed using Affymetrix 219 Gene Array analysis (Affymetrix, Santa Clara, CA, USA). For selection of candidate genes, probes with the greatest log-fold change between phenotypes were selected. Genes found to be highly expressed in GBM cell line G48a were excluded in order to avoid factors present in both macrophage and tumor cells [6]. 

### 2.8. Immune Cell Isolation

All human samples were obtained from the Brain Tumor Center of Excellence in the Wake Forest Baptist Medical Center Comprehensive Cancer Center and handled according to Wake Forest IRB protocol #8427. GBM patient tumors were minced and dissociated in a collagenase dissociation buffer. Cells were separated from debris using centrifugation in a Ficoll gradient. The cell layer was washed twice with PBS and counted. Tissue-derived cells were counted prior to freezing in Neurobasal media with 5% DMSO until submission for single-cell sequencing. Immediately prior to sample submission, dissociated tumor cells were thawed, and washed with PBS prior to incubation with CD45-conjugated MACS magnetic beads (Miltenyi Biotech, Auburn, CA, USA). Bound cells were passed through a mini-MACS magnetic bead isolation column and washed twice with MACS buffer. CD45^+^ cells were eluted, counted, and submitted for single-cell RNA sequencing. 

### 2.9. Single-Cell RNA Sequencing

Samples prepared as described above were submitted to the Cancer Genomics Shared Resource in the Wake Forest Baptist Medical Center Comprehensive Cancer Center for single-cell sequencing and analysis. Samples with ≥60% viable cells were processed for cDNA library construction using the 10× Genomics Chromium platform (10× Genomics, Pleasanton, CA, USA) with v3 chemistry. Indexed libraries were paired-end sequenced on an Illumina NovaSeq 6000 (Illumina, San Diego, CA, USA) targeting 2500 cells per sample at a median read depth of 100,000 reads per cell. Raw bcl files were converted to fastq for read demultiplexing, alignment, and counting using the CellRanger toolkit (10× Genomics, Pleasanton, CA, USA). Data QC parameters were applied as previously described [34] to select high-quality cellular transcriptomes. Data dimensionality reduction (t-SNE) and clustering (K-means) algorithms were used to assess cell-to-cell relationships. Immune cell identities were assigned as previously described [34]. Cross-sample cell populations were compared for differences in gene expression using negative binomial models with FDR correction.

### 2.10. Western Blot

Preparation of cell and tumor lysates and Western blot analysis were performed as described previously [35]. Tumor specimens were obtained from the Brain Tumor Center of Excellence at the Wake Forest Baptist Medical Center Comprehensive Cancer Center. For analysis of polarized THP1 cells, nonadherent cells were collected by centrifuging media from each well and combining the pellet to the respective population of adherent cells prior to addition of lysis buffer. 

### 2.11. Immunostaining

All human tissue samples were obtained from the Tumor Bank of the Comprehensive Cancer Center at Wake Forest University/Brain Tumor Bank of the Brain Tumor Center of Excellence. Specimens were collected and handled according to the Wake Forest IRB protocol #8427. Immunohistochemistry and immunofluorescence were performed as described previously [36]. Photomicrographs were taken at 20× on an Olympus IX70 microscope (Olympus, Center Valley, PA, USA) using either a Retiga 2000R camera (Media Cybernetics, Silver Springs, MD, USA) with ImagePro Plus (ver 5.1, Media Cybernetics, Silver Springs, MD, USA) software or an Olympus DP80 camera (Olympus, Center Valley, PA, USA) with CellSense software (ver 2.3, Olympus, Center Valley, PA, USA).

### 2.12. TCGA Analysis

The Cancer Genome Atlas (TCGA) Glioblastoma (GBM) and Lower Grade Glioma (LGG) harmonized RNAseq data sets were downloaded from the Genomics Data Commons Data Portal (https://portal.gdc.cancer.gov/, accessed on 30 August 2021) and FPKM-UQ normalized using the Python *gdc-rnaseq-tool* script (https://github.com/cpreid2/gdc-rnaseq-tool, accessed on 30 August 2021). Non-malignant tissues were omitted from the analysis. *ALDH1A2* expression distributions were analyzed in primary and recurrent tumor tissues separately. Pairwise statistical comparisons were performed using a Wilcoxon rank-sum test.

### 2.13. Statistical Analysis

Each sample was normalized to the actin loading control for that sample. The two-sample *t*-test was used to compare the normalized measures between two treatment groups. Analysis of variance was used for comparisons among more than two treatment groups. The one-sample *t*-test was used to compare the mean of normalized measures in one group to a specific known value. Since primary and recurrent tumors were collected from the same patient, we used the paired *t*-test to examine whether the mean difference in expression of CD163 or ALDH1A2 between primary and recurrent tumors was different from 0. A *p* value of <0.05 was considered statistically significant.

## 3. Results

### 3.1. M2 Macrophages in GBM Specimens

**Figure 1:**
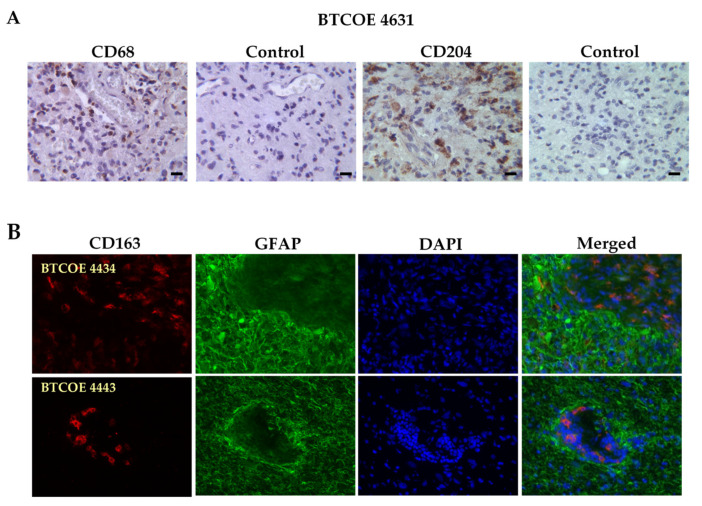
An abundance of M2 macrophages in GBM tumors. (**A**) BTCOE 4631 GBM sample stained for the pan-macrophage marker CD68 and the M2 macrophage marker CD204. (**B**) Representative images of the M2 macrophage marker CD163 (red), and the tumor cell marker GFAP (green) in GBM patient samples shows the presence of M2 macrophages both within tumors and the tumor vasculature. Scale bars for all images are 50 µm. These images are representative of over 10 patient tumor specimens tested.

Macrophages have been shown to infiltrate GBM tumors and in fact, up to 30% of GBM mass can be represented by macrophages and microglia [8,21]. We analyzed the presence of macrophages and microglia in the GBM specimens in our laboratory using the pan-macrophage/microglial marker CD68. As M2 macrophages, unlike M1 macrophages, are recognized to be tumor supporting [21], we utilized the M2 markers CD204 and CD163 to investigate the presence of M2 macrophages in the GBM tumor microenvironment. As expected, the macrophages were abundantly present within the GBM tumor microenvironment (Figure 1). In addition, CD163^+^ macrophages were also present in the tumor vasculature itself as shown in BTCOE 4443 and in agreement with our previous data (Figure 1B) [6]. 

### 3.2. M2 Macrophages Highly Express ALDH1A2 In Vitro

**Figure 2:**
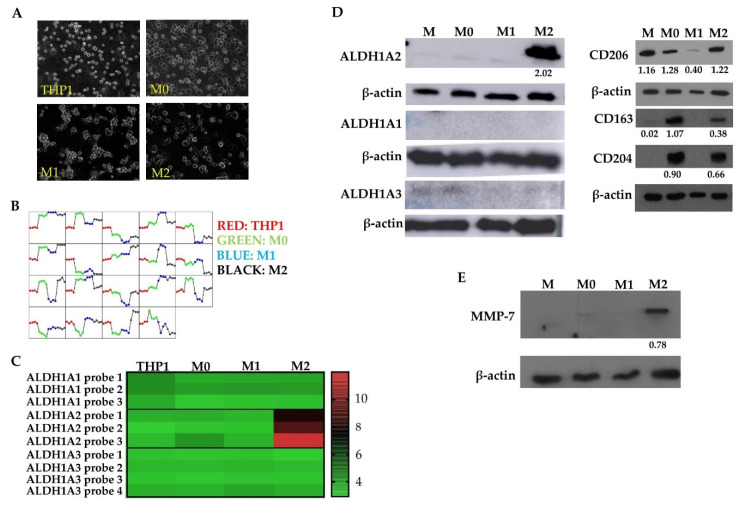
M2 macrophages highly express ALDH1A2 in a THP1 cell model system. (**A**) THP1 monocytic cells were differentiated to activated (M0), M1, and M2 states reflected by changes in morphology. (**B**) Comparison of dominant gene expression patterns between THP1 macrophage polarization states. (**C**) *ALDH1A2* upregulation in M2 macrophages compared to other activation or polarization states (*p* < 0.0001). Other ALDH1 family genes, *ALDH1A1* and *ALDH1A3* showed low to no expression in THP1 cells regardless of activation or polarization state. Results are representative of 4 arrays. Western blot of (**D**) ALDH1A2, ALDH1A1, ALDH1A3, CD206, CD163, CD204, (representative of at least 3 blots) and (**E**) MMP-7 (representative of 2 blots) immunoreactive proteins in THP1 cells (M: naïve cells, M0: activated with PMA, M1: activated, polarized with LPS, IFNγ, M2: activated, polarized with IL-4, IL-13). MMP-7 is only expressed in M2 macrophages in agreement with the gene expression array. Normalized densitometry is reported below the Western blot.

In order to further characterize M2 macrophages in the context of GBM, we utilized the THP1 monocytic cell model system, which has been used as a macrophage model and can be activated and polarized to the M1 or M2 state (Figure 2A,D) [32,37]. A gene expression array was performed on THP1 cells which were first activated with PMA (termed M0) then pharmacologically differentiated to the M1 state, with LPS and IFNγ, or to the M2 state, with IL-4 and IL-13. Genes that were found to be highly expressed in GBM tumor cells [6] were subtracted from the analysis in order to identify macrophage-specific factors. The consistency of our data was evaluated by assessing the expression of groups of genes across each replicate. We found that gene expression was similar across all replicates in all groups of genes tested (Figure 2B). This array produced a list of genes including *ALDH1A2*, *SerpinB3*, and *SerpinB4* in M2 polarized THP1 cells; *SerpinG1*, *SerpinB7*, and *P2RX7* were upregulated in M1 polarized THP1 cells. We focused on *ALDH1A2* which showed up to 8-fold higher expression in M2 polarized THP1 cells compared to M1 polarized or activated THP1 cells (Figure 2C). 

To validate the gene expression analysis, Western blots demonstrated that M2 THP1 cells highly express immunoreactive ALDH1A2 in comparison to untreated, activated (M0), or M1 polarized THP1 cells (Figure 2D). Additionally, we assessed the expression of two other common ALDH1 family proteins whose function is also to catalyze retinoic acid synthesis: ALDH1A1 and ALDH1A3. THP1 cells did not express either ALDH1A1 or ALDH1A3 regardless of activation or polarization state (Figure 2D; left panel). This indicated the specificity of the ALDH1A2 over-expression in the M2 polarized THP1 cells. Furthermore, polarization of THP1 cells was evaluated by assessing expression of M2 macrophage markers CD206, CD204, and CD163, each of which were found in M2 polarized THP1 cells (Figure 2D; right panel). Further validation of gene expression array results was achieved by performing a Western blot to assess expression of MMP-7, which was also found to be differentially expressed in M2 polarized THP1 cells compared to other treatment groups. The MMP-7 expression was higher in the M2 polarized THP1 cells indeed when compared to untreated (M), activated (M0), or M1 polarized THP1 cells (Figure 2E). These data suggest that ALDH1A2, and also MMP-7, are associated with the M2 polarization state in a human monocytic cell model.

**Figure 3:**
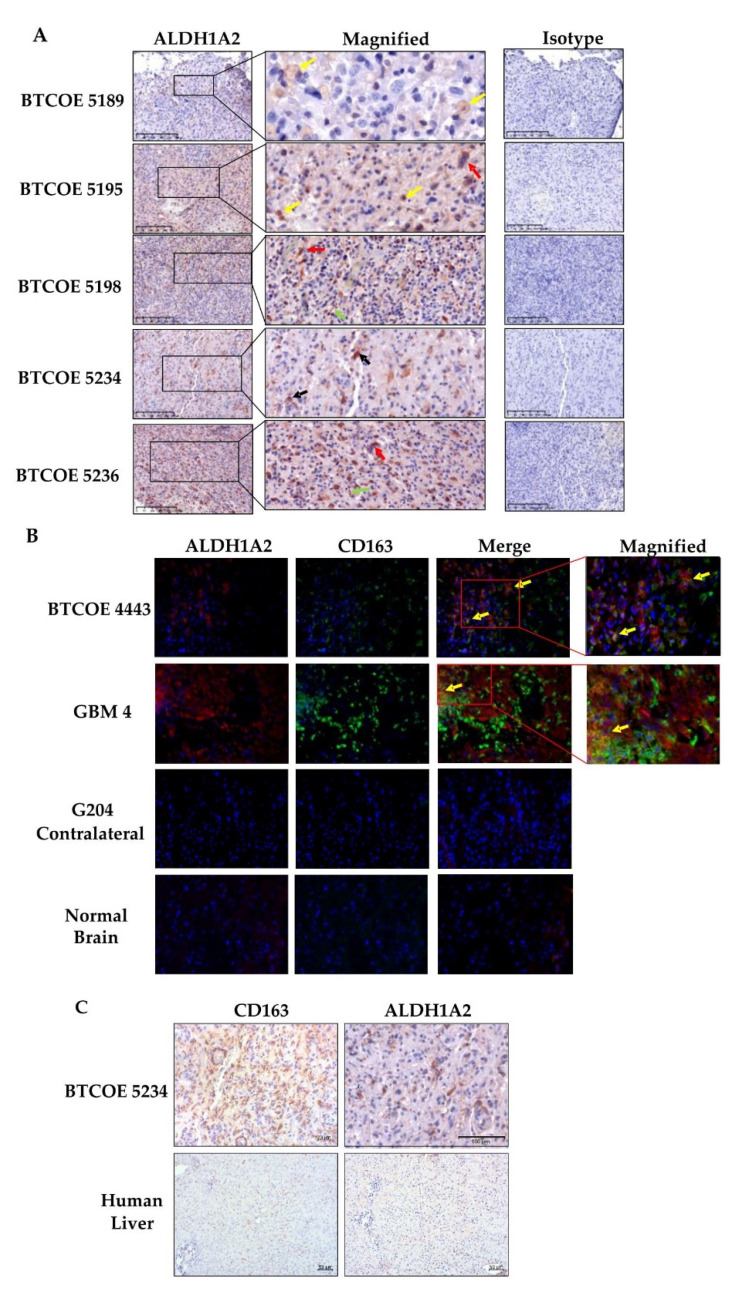
Expression of ALDH1A2 in the GBM tumor microenvironment. (**A**) Immunohistochemical staining of tumors used for single-cell sequencing (Figure 6 below) shows a relatively high number of ALDH1A2^+^ cells. These cells are represented by macrophages (yellow arrows), multinucleated tumor cells (red arrows), non-activated microglia (green arrows), and reactive astrocytes (black arrows). These images are representative of over 10 tumors tested. (**B**) Immunofluorescent staining for ALDH1A2 and the M2 macrophage marker CD163. Some M2 macrophages are also ALDH1A2 positive (yellow arrows). Please note that neither CD163 nor ALDH1A2 are abundant in normal brain. These images are representative of over 10 tumors tested. Scale bars are 50 µm. (**C**) ALDH1A2 is expressed in GBM-associated macrophages but not in normal human liver, which is enriched in CD163^+^ macrophages.

To explore ALDH1A2 expression in GBM specimens, we performed immunohistochemical staining to assess both ALDH1A2 and CD163. GBM tumors expressed ALDH1A2 in macrophages and non-activated microglia, (Figure 3A; five representative samples are shown). We also examined co-localization of ALDH1A2^+^ and CD163^+^ cells within GBM tumors by fluorescent staining. While some CD163^+^ cells were also ALDH1A2^+^, there also existed a population of ALDH1A2^+^/CD163 cells as well as ALDH1A2^−^/CD163^+^ cells (Figure 3B). This indicates that ALDH1A2^+^ cells make up a subpopulation of cells in the GBM tumor microenvironment and do not encompass all M2 GAM. Of interest, neither CD163 nor ALDH1A2 were detected in normal brain sections, suggesting that ALDH1A2 expression takes place within the GBM tumor microenvironment and is not a function of the non-diseased brain (Figure 3B). Next, we evaluated ALDH1A2 expression in normal liver as the liver is known to have resident macrophages called Kupfer cells. These macrophages are present in both M1 and M2 activation states [38]. While many CD163^+^ cells were present within normal liver sections, they did not stain for ALDH1A2 at all (Figure 3C). This is similar to GBM where ALDH1A2 is not restricted to CD163^+^ M2 macrophages and may indicate a disease-specific phenotype of macrophages. 

### 3.3. ALDH1A2 Is Highly Expressed in GBM Compared to LGG and Other ALDH1 Family Proteins

**Figure 4:**
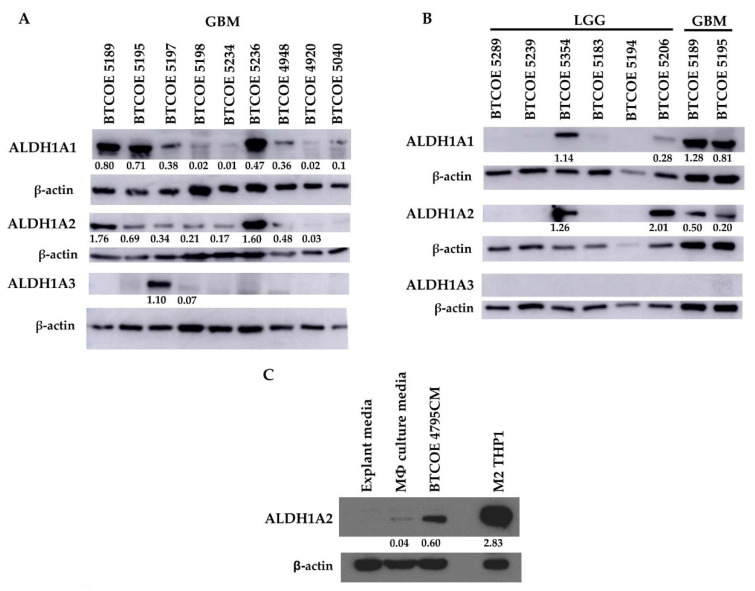
ALDH1A2 is expressed at higher levels in GBM compared to LGG and other common ALDH1 family proteins. Expression of ALDH1A1, ALDH1A2, and ALDH1A3 in (**A**) GBM patient samples (representative of at least 2 blots), and (**B**) low-grade glioma (LGG) (representative of 5 blots). (**C**) GBM cell-conditioned media is sufficient to induce ALDH1A2 expression in the THP1 monocytic cell model (representative of 2 blots).

As mentioned above, there are two other ALDH1 family proteins, namely ALDH1A1 and ALDH1A3. Of these two, ALDH1A1 has been reported as a stem cell marker [39,40,41], while ALDH1A3 appears to have more tissue-specific expression, being highly expressed in the kidney, salivary glands, stomach, and breast [40]. These two genes were not expressed in our THP1 gene expression array (Figure 2C) and their corresponding proteins were absent in THP1 cells regardless of treatment or polarization state (Figure 2D). We evaluated ALDH1 family protein expression in a panel of GBM tumor lysates. ALDH1A1 was expressed in six out of nine tumors; ALDH1A2 was also expressed in six out of nine tumor samples, and ALDH1A3 was present in only one out of nine tumors examined (Figure 4A). When assessing ALDH1 family proteins in low-grade glioma (LGG), a panel of six LGG samples showed that two samples expressed ALDH1A1 and two samples also expressed ALDH1A2, though to varying degrees. ALDH1A3 was not detected in the panel of six LGG samples (Figure 4B). Taken together, these data show that ALDH1A2 is more prevalent in GBM when compared to LGG. Additionally, ALDH1A2 and ALDH1A1 appear to be the primary ALDH1 family proteins expressed in GBM, while ALDH1A3 does not appear to be commonly expressed in either GBM or LGG. 

Having determined that cells within the GBM tumor microenvironment express ALDH1A2, we wanted to examine whether GBM cells influence the expression of ALDH1A2 in monocytic cells. Naïve THP1 cells were cultured under normal conditions and in conditioned media (CM) from BTCOE 4795 GBM cells. CM from GBM cells was able to increase ALDH1A2 expression in naïve THP1 cells (Figure 4C), indicating that crosstalk between GBM cells and macrophages induces ALDH1A2 expression.

**Figure 5:**
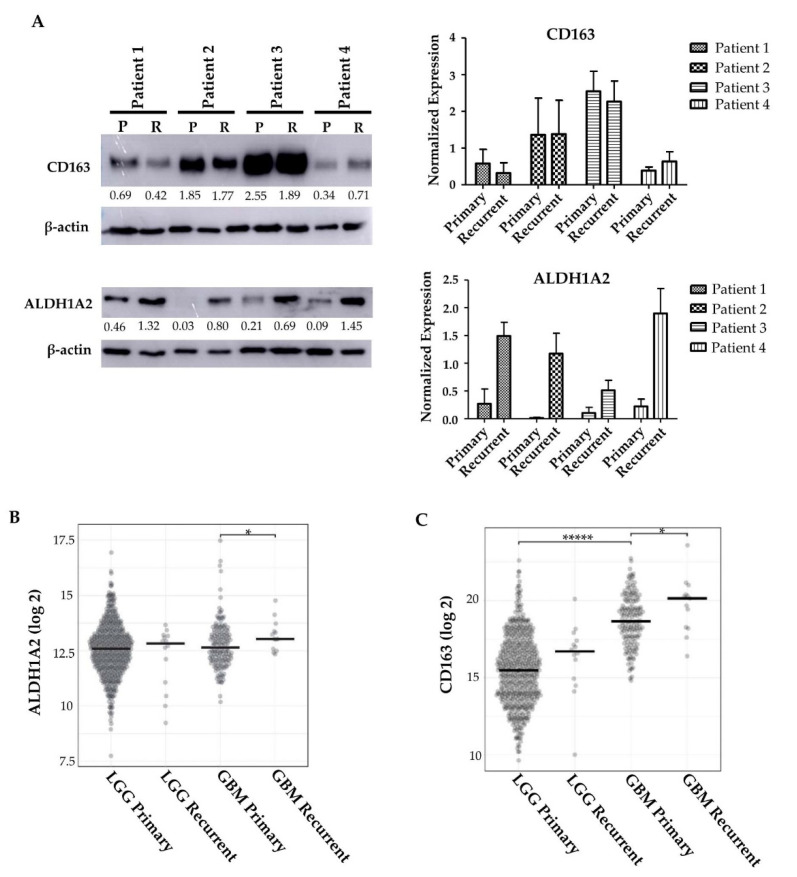
ALDH1A2 expression changes upon tumor recurrence. (**A**) ALDH1A2 and CD163^+^ expression in the recurrent tumors of 4 matched patient tumors (representative of at least 2 blots). (**B**) TCGA analysis of ALDH1A2 gene expression in LGG and GBM primary and recurrent tumors. * indicates *p* = 0.048. (**C**) TCGA analysis of CD163 gene expression in LGG and GBM primary and recurrent tumors. ***** indicates *p* < 0.0001, * indicates *p* = 0.036.

As therapeutic interventions affect the tumor microenvironment [42,43], we next asked whether the expression of ALDH1A2 was changed upon tumor recurrence. To investigate this, we performed Western blots on matched patient primary and recurrent tumor samples. The expression of ALDH1A2 increased upon recurrence (Figure 5A). Interestingly, this increase does not appear to be linked to CD163 expression, as CD163 expression appears to not change or decrease upon tumor recurrence (Figure 5A). To expand this analysis, we queried The Cancer Genome Atlas (TCGA) to determine if gene expression of *ALDH1A2* and *CD163* change in recurring tumors. We found that *ALDH1A2* gene expression is significantly increased upon GBM recurrence, a result which is reflected by our Western blot (Figure 5A,B). In LGG, there is a trend toward increased ALDH1A2 expression upon tumor recurrence, though this did not achieve statistical significance. *CD163* gene expression was significantly increased in GBM when compared to LGG. Interestingly, CD163 gene expression was also significantly increased upon tumor recurrence in GBM (Figure 5C). This increase in gene expression does not appear to translate to an increase in protein expression (Figure 5A). We stained primary and recurrent GBM samples for CD163 and observed a higher CD163^+^ population in the primary tumors than the recurrent tumor samples, in line with our Western blot results (data not shown). The discordant result between gene expression and protein expression may be due to an increased degradation of CD163 or limited transcript availability. It is tempting to speculate that ALDH1A2 might be a biomarker of GBM recurrence and its progression.

### 3.4. ALDH1A2 Is Expressed in the Microenvironment of GBM Tumors

**Figure 6:**
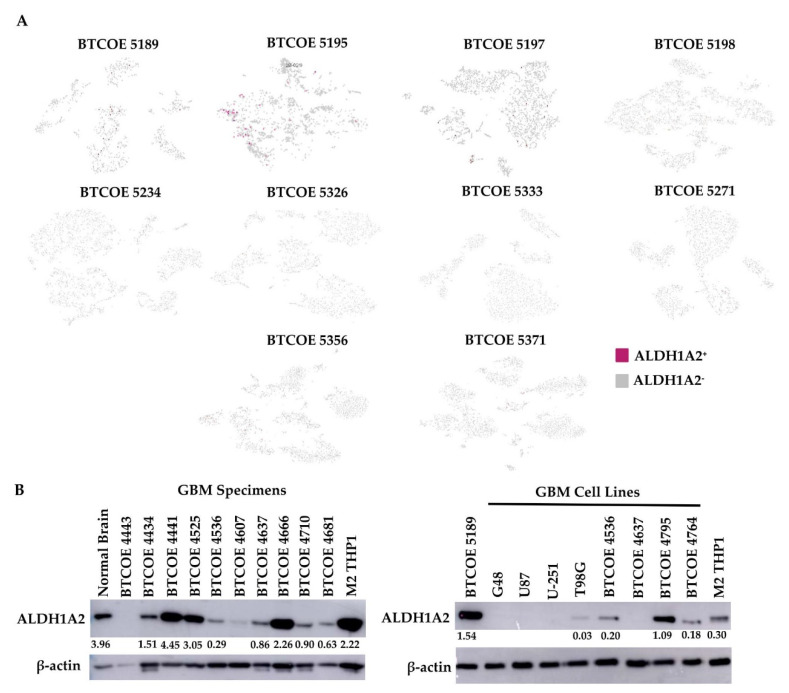
ALDH1A2 expression in GBM tumors. (**A**) Single-cell sequencing was performed on the CD45^+^ immune infiltrate of GBM tumors. (**B**) Expression of ALDH1A2 in lysates of GBM tumors (left panel) and in a panel of GBM cell lines (right panel) (representative of at least 3 blots).

As ALDH1A2 is expressed in GAM, we evaluated the expression of ALDH1A2 in the immune infiltrate of ten GBM specimens. We performed single-cell RNA sequencing of CD45^+^ cells isolated from GBM. Having identified the different cell populations in each patient immune infiltrate sequenced, we found that a low number of monocytic cells were *ALDH1A2^+^* (Figure 6A), a result which was surprising in view of the gene expression array performed on the THP1 cell model or the protein expression by Western blots and immunohistochemistry of the sequenced tumor samples (Figure 3A and Figure 4A). This may indicate that the RNAs for ALDH1A2 are low to moderately expressed and may have a short half-life relative to their corresponding protein levels, which were easily detectable. Therefore, we again stained for *ALDH1A2* immunoreactivity in 10 GBM tumor specimens and 8 cell lines and found that ALDH1A2 was expressed quite prominently in GBM specimens (Figure 6B, left panel). Interestingly, ALDH1A2 was largely absent in established GBM cell lines and moderately expressed in patient-derived low-passage cell lines (Figure 6B, right panel), suggesting that the expression of ALDH1A2 in established GBM tumor cells is not preserved under in vitro culture conditions.

### 3.5. Retinoic Acid Contributes to the Invasive Potential of GBM Tumor Cells via Modulation of MMP-2 and MMP-9 in Monocytic Cells

**Figure 7:**
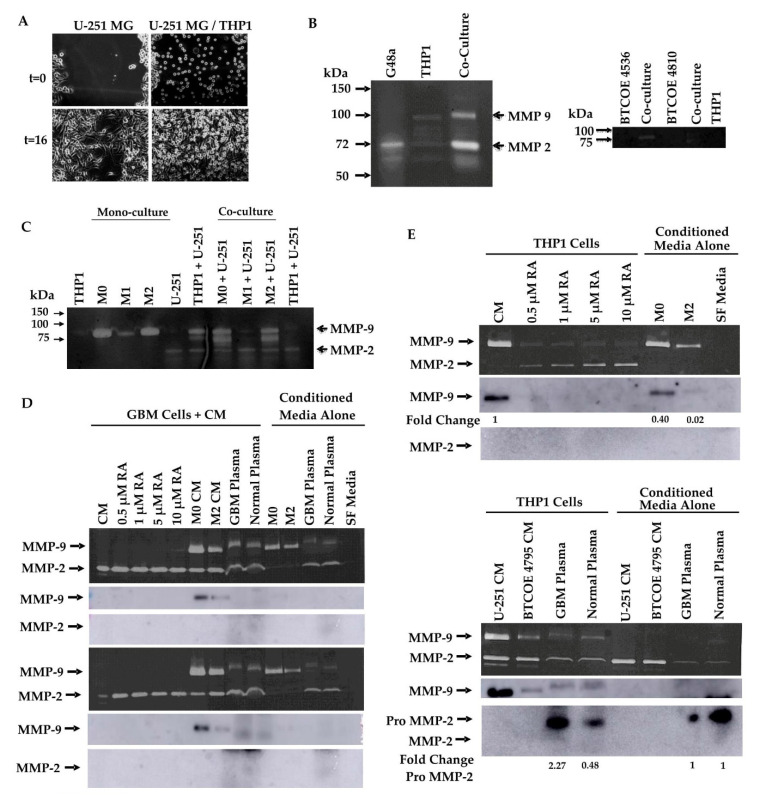
Retinoic acid modulates expression and activity of MMP-2 and MMP-9. (**A**) Scratch wound assay of GBM cells co-cultured with THP1 monocytic cells. Monoculture showed 74% wound closure while co-culture showed 91% closure (*p* < 0.0001). Gelatin zymograms for MMP-2 and MMP-9 activity performed on (**B**) media from GBM cells, macrophages, and their co-culture, (**C**) differentiated THP1 (M: naïve, M0: activated) cells in monoculture and co-cultured with GBM cells, (**D**) GBM cells treated with increasing concentrations of RA, conditioned media from differentiated THP1 macrophages, and human plasma, (upper panel: U-251; lower panel: BTCOE 4795). MMP-2 activity in BTCOE 4795 cells was significantly increased in 0.5 uM RA (*p* = 0.0002) and 1 uM RA (*p* = 0.003) treatment groups by one-sample *t*-test. MMP-9 activity in BTCOE 4795 cells was significantly increased in M0 CM treatment group (*p* = 0.03). Zymogram of (**E**) THP1 cells treated with RA, conditioned media from GBM cells, and human plasma. MMP-9 activity was significantly decreased in each RA treatment group (*p* = 0.0004, *p* = 0.005, *p* = 0.004, *p* = 0.007, respectively) and in M2 CM (*p* = 0.03). M2 CM also showed significantly decreased MMP-9 activity compared to M0 CM (*p* = 0.03). Zymograms and blots are representative of at least 2 experiments performed.

Macrophages have been shown to have a role in many aspects of GBM progression [21]. To examine how interactions with macrophages influence cell migration, we employed a scratch wound-healing assay on GBM cells co-cultured with THP1 cells. Co-culture with THP1 cells increased the rate at which GBM cells are able to close the wound after 16 h by significantly increasing the percentage of wound closure from 74% in monoculture to 91% in co-culture (Figure 7A). 

To examine how interactions between GBM cells and macrophages modulate markers of invasiveness, we utilized gelatin zymography to examine MMP-2 and MMP-9 activity. G48a GBM cells were cultured with or without THP1 cells and the activity of MMP-2 and MMP-9 appeared to be increased in THP1 cells co-cultured with G48a GBM cells (Figure 7B, left panel). A similar result is seen when patient-derived GBM cells are cocultured with THP1 cells (Figure 7B, right panel). To further elucidate the effect different macrophage polarization states would have on this activity, THP1 cells were pharmacologically polarized and co-cultured with U-251 GBM cells. Both activated (M0) and M2 polarized THP1 cells increased MMP-2 and MMP-9 activity in co-culture while M1 macrophages co-cultured with U-251 cells demonstrated no increase in MMP activity above U-251 media alone (Figure 7C). 

GAM express ALDH1A2 (Figure 3), an enzyme whose function is to produce retinoic acid from retinaldehyde. RA has also been reported to modulate MMP-2 and MMP-9 expression *in vitro* in a few cell lines, including T98G GBM cells, THP1 cells, and A375 melanoma cells [44,45,46]. We asked whether RA could be responsible for the changes in MMP-2 and MMP-9 activity observed in both monocultures of THP1 cells and co-cultures of THP1 cells with GBM cells (Figure 7B,C). To this end, gelatin zymography was used to first examine the ability of RA to modulate the activity of MMP-2 and MMP-9 in GBM cells. U-251 (Figure 7D, upper panel) and BTCOE 4795 GBM cells (Figure 7D, lower panel) were treated with increasing concentrations of RA. RA up to 10 µM did not appear to change the activity of MMP-9 in both U-251 and BTCOE 4795 GBM cells; however, in U-251 cells, a small increase in MMP-9 activity was observed at 10 µM RA (Figure 7D). Additionally, while activity of MMP-2 did not increase in U-251 cells, it did significantly increase upon treatment with 0.5 and 1 µM RA in BTCOE 4795 cells with p values of 0.0002 and 0.003, respectively (Figure 2D). In addition, GBM cells treated with activated (M0) or M2 polarized THP1-conditioned media appeared to show a selective increase in MMP-9 activity (Figure 7D). A slight increase in MMP-9 activity was observed when serum-free media was supplemented with 10% plasma from either a normal control or GBM patient. This increase appeared to be due to factors present within the plasma rather than solely an induced response, as the increase was not significant (Figure 7D). 

We asked whether changes in MMP-2 or MMP-9 activity were due to changes in protein expression of these metalloproteinases. While the GBM cells did not appear to secrete either MMP-2 or MMP-9 into the media upon treatment with increasing concentrations of RA or human plasma, culturing GBM cells in activated (M0) or M2 polarized THP1 CM increased the level of immunoreactive MMP-9 (Figure 7D). 

While RA did not appear to largely modulate the activity of MMP-2 or MMP-9 from GBM cells, it may have an effect on these enzymes’ activity in THP1 cells. THP1 cells were treated with up to 10 µM RA, GBM CM, normal plasma, or GBM patient plasma. Untreated THP1 cells had measurable MMP-9 activity; however, this activity was significantly reduced upon treatment with RA at concentrations as low as 0.5 µM (Figure 7E, upper panel). Additionally, the activity of MMP-2 increased in a concentration-dependent manner in THP1 cells treated with RA, though this result did not achieve statistical significance (Figure 7E, upper panel). Interestingly, M2 polarized THP1 cells showed significantly lower MMP-9 activity when compared to untreated or activated (M0) THP1 cells (Figure 7E, upper panel). As these cells exhibited higher ALDH1A2 expression, the decrease in MMP-9 activity may be due to an increase in RA. Addition of GBM CM or plasma caused a small increase in MMP-2 activity, though this appeared to be due to factors present in the CM rather than an induced response in THP1 cells. When secreted protein levels of MMP-2 and MMP-9 in these samples were assessed, secretion of MMP-9 was diminished in THP1 cells treated with RA, and was decreased in M2 polarized THP1 cells. Secreted MMP-2 was below the level of detection in THP1 cells treated with RA and in the conditioned media of activated or M2 polarized THP1 cells (Figure 7E, upper panel). Secreted MMP-2 and -9 were below the level of detection in THP1 cells treated with GBM CM, but there was an increase in the pro-form of MMP-2 in THP1 cells treated with human plasma (Figure 7E, lower panel). These results suggest that RA modulates MMP expression and subsequent activity in macrophages, but not the GBM cells.

## 4. Discussion

The cells of the GBM tumor microenvironment, such as GAM, have been shown to play important roles in angiogenesis, immune suppression, and tumor progression [47]. Understanding the mechanisms behind these actions, as well as understanding the specific factors that differentiate GAM from other macrophages, is important for targeting potential therapeutics as well as for discovering specific markers identifying GAM. There is an abundant presence of macrophages, including the alternatively activated M2 macrophage, in GBM. We performed a gene expression array and identified macrophage-related markers in the THP1 monocytic cell model system. Of these, ALDH1A2, an enzyme that catalyzes the synthesis of retinoic acid, was highly expressed in GBM microenvironment cells. Specifically, ALDH1A2 is expressed in macrophages and non-activated microglial cells, and also multi-nucleated tumor cells and reactive astrocytes. In this study, we explored the expression of ALDH1A2 in GAM and how RA can influence the progression of GBM via changes in MMP expression and activity in the studied cells. 

While the ALDH1A2 gene is over-expressed in a rather low number of CD45^+^ monocytic cells in the GBM microenvironment, protein expression is considerably more prominent by Western blot, IHC, and IF. This is related to the fact that, at least in part, not only macrophages over-express this enzyme. While ALDH1A1 is expressed in GBM, it is also expressed in other cancer types according to previous reports [39,40,41]. Interestingly, ALDH1A2 expression is virtually undetectable in GBM cells in culture, which further supports that the tumor microenvironment influences the expression of ALDH1A2 in GBM tumor cells. 

In addition, ALDH1A2 expression is higher in recurring tumors. This phenomenon is not linked to the CD163^+^ M2 macrophage population. Thus, an increase in ALDH1A2 immunoreactivity may be reflective of a change in a different subpopulation of macrophages. Reactive astrocytes, non-activated microglia, and multi-nucleated tumor cells also stained for ALDH1A2. It is possible that one or more of these cell populations could be responsible for the increase in ALDH1A2 expression upon tumor recurrence, though this has yet to be fully investigated. Our results suggest the hypothesis that other cell types may be responsible for this increase in ALDH1A2 expression, as cells which co-stain for GFAP, a tumor cell, and reactive astrocyte markers were found in GBM tumors (data not shown). 

We showed that macrophages increase the migration of GBM cells as well as increase invasive potential via an increase in MMP activity. Mechanistically, this could occur through the ALDH1A2-mediated synthesis of RA from retinaldehyde. RA modulates both MMP-9 and MMP-2 expression and activity in THP1 cells, downregulating MMP-9 in naïve THP1 cells even at low concentrations, but upregulating MMP-2. The reduction in MMP-9 in M2 polarized THP1 cells could be due to the increase in ALDH1A2 expression found in these cells when compared to activated or naïve THP1 cells. RA did not appear to have an effect on GBM cells, however. This would suggest that the function of RA is primarily to modulate the tumor microenvironment via effects on those cells instead of a direct effect on tumor cells.

Retinoic acid has been explored as a treatment for various different tumor types, including oral, head and neck, breast, and GBM. Toxicity associated with RA therapies has led to the development of other retinoid compounds or therapeutics which target different pathways influenced by retinoic acid [26]. The use of such strategies has had inconsistent effects in the treatment or prevention of solid tumors, although all-trans-RA (atRA) has proven successful in the treatment of acute promyelocytic leukemia [26]. 

In regard to GBM, RA has been used to treat various GBM cell lines as well as mouse xenografts with limited and inconsistent effect. In some instances, treatment with RA led to inhibited proliferation and migration of GBM stem-like cells [27,28]. However, in other cell lines, RA increased proliferation and did not affect differentiation and apoptosis. In the clinic, single-agent treatment with RA produced no activity in recurrent GBM [26]; in a phase II clinical trial, a retinoid combined with TMZ did not increase progression-free survival in primary GBM patients [48]. This is to say that use of RA as a therapeutic has met with little success in GBM. This is likely due to the complexity of RA signaling, and indicates that general use of RA as a sole therapeutic is not a viable option for GBM treatment. We have shown that macrophages in the GBM microenvironment express higher levels of ALDH1A2 and that RA modulates MMP-2 and MMP-9 expression and activity; this could lead to downstream modulation of tumor invasiveness and progression in a local fashion and may explain the lack of therapeutic benefit of RA. 

Our data show that GBM cells are resistant to the effects of RA on modulating MMP-2 and MMP-9 activity. The idea that GBM cells resist RA signaling is supported by a study in which RA was shown to be sequestered in the cytoplasm of GBM cells, and was therefore unable to induce transcriptional changes [49]. However, we show that macrophages are responsive to RA and that treatment with RA induces increased activity of MMP-2 while decreasing activity of MMP-9, which may aid in tumor progression. It is possible, therefore, that RA exerts a stimulatory influence on the GBM tumor microenvironment, which allows for promotion of the tumor via increased infiltration into the surrounding brain parenchyma. One may hypothesize that GBM can take advantage of RA signaling effects on microenvironment cells such as GAM in order to progress, while itself remaining resistant to the anti-tumor effects RA signaling may have on tumor cells. 

This study only covers one facet of the extraordinarily complex GBM tumor microenvironment: GBM-associated macrophages. In the future, it will be important to understand how the expression of ALDH1A2 in reactive astrocytes and multi-nucleated tumor cells affects the GBM tumor cells and the microenvironment. RA has effects on a variety of different signaling pathways; this study was limited to its effect on GAM and specifically on how GAM promote GBM progression. A better understanding of the effect RA has on GBM and its microenvironment is important for further understanding the effect RA and its downstream pathways have on tumor progression. This may also lead to the identification of new targets to abrogate these effects.

## 5. Patents 

“Detection of Malignancy in Brain Cancer” (WO2015070197A1).

## Data Availability

Data presented in this study are contained within the article or obtained from TCGA as described in Materials and Methods.
